# Care-engaged individuals with polysubstance use in Northeastern US are undertreated for methamphetamine use disorder: a retrospective cohort study

**DOI:** 10.1186/s13722-021-00267-1

**Published:** 2021-09-26

**Authors:** Mimi Yen Li, George A. Alba, Julian Mitton, Benjamin Bearnot

**Affiliations:** 1 Harvard Medical School, 25 Shattuck St, Boston, MA 02115 United States; 2 CommonSpirit Heath Enterprise Population Health, 185 Berry Street, Suite 200, San Francisco, CA 94107 United States; 3 Department of Medicine, Massachusetts General Hospital, 100 Cambridge Street, Suite 1600, Boston, MA 02114 United States

**Keywords:** Methamphetamine, Opioid crisis, Harm reduction, Stimulants, Drug overdose

## Abstract

**Background:**

Stimulant use has increased across the US, with concomitant opioid and methamphetamine use doubling between 2011 and 2017. Shifting patterns of polysubstance use have led to rising psychostimulant-involved deaths. While it is known that individuals who use methamphetamine require greater access to treatment, there is still little known about methamphetamine use and treatment among individuals who are already engaged in outpatient substance use treatment.

**Objectives:**

To characterize care-engaged individuals who use methamphetamine to guide harm reduction and treatment strategies.

**Methods:**

Retrospective cohort study of individuals at a large academic medical center in Massachusetts with ≥ 2 positive methamphetamine oral fluid toxicology tests between August 2019 and January 2020. We performed descriptive analysis of sociodemographic, medical, and drug use characteristics and a comparative analysis of injection methamphetamine use versus other routes of use.

**Results:**

Included were 71 individuals [56 male (80%), 66 non-Hispanic white (94%), median age 36 (IQR 30–42)]. Nearly all had opioid (94%) and stimulant use disorder (92%). Most had (93%) or were (83%) being treated with medications for opioid use disorder, but few received pharmacologic treatment for methamphetamine use disorder (24%). None received contingency management treatment.

People who inject methamphetamine (68%) were more likely to have a history of overdose (91% vs. 70%; p = 0.02), have HCV (94% vs. 52%; p < 0.01), use fentanyl (93% vs. 65%; p = 0.02), and engage in sex work (19% vs. 0%; p = 0.03) compared to those who used via other routes. Both groups had prevalent homelessness (88% vs. 73%; p = 0.15), incarceration (81% vs. 64%; p = 0.11), depression (94% vs. 87%; p = 0.34), and bacteremia (27% vs. 22%; p = 0.63).

**Conclusions:**

Individuals in our study had high prevalence of polysubstance use, particularly concomitant methamphetamine and opioid use. Individuals who were well connected to substance use treatment for their opioid use were still likely to be undertreated for their methamphetamine use disorder and would benefit from greater access to contingency management treatment, harm reduction resources, and resources to address adverse social determinants of health.

## Introduction

Stimulant use has escalated in the United States (US), following three earlier waves of prescription opioid, heroin, and fentanyl use, to forge a fourth wave of the opioid crisis [1–3]. A rise in amphetamine-related serious bacterial infections and hospitalizations has contributed to a nearly five-fold increase in amphetamine-related hospital costs [4, 5]. Overdose deaths associated with amphetamine-type stimulants have increased approximately 30% per year between 2012 and 2018, with methamphetamine accounting for 11% of the total number of US overdose deaths in 2011 [6]–[9]. The doubling of concomitant opioid and methamphetamine use between 2011 and 2017 suggest the rise of methamphetamine is connected to, not separate from, the opioid crisis [1]. Convergence of the opioid and methamphetamine crises poses particular risks in the Northeastern US, where opioids such as fentanyl, heroin, and oxycodone are among the top five most common drugs involved in overdoses [10]. Dynamic changes in drug hotspot patterns already suggest methamphetamine use, which have historically predominated in the Western US, is no longer a regional issue [11]. From 2008 to 2017, primary heroin treatment admissions involving methamphetamine use increased in all US geographic regions, with a thirteen-fold increase in the Northeast [12]. Rising concomitant methamphetamine use has led to treatment challenges as individuals who use methamphetamine are less likely to be retained in opioid use treatment [13]. In 2015, 36% of opioid-related overdoses in Massachusetts involved stimulants [9]. From 2018 to 2019, the largest relative increase in the psychostimulant-involved death rate occurred in the Northeast (44%) [14]. Emergency departments and outpatient settings in the Northeast have also begun to see a rise in methamphetamine use in patients [8, 15].

However, there is limited characterization of individuals who use methamphetamine in the Northeastern US, and particularly for subpopulations who use methamphetamine via injection, which has been correlated with higher prevalence of infectious disease, psychiatric conditions, and adverse social determinants of health [16–18]. Moreover, while a number of studies highlighting the medical and social complexity of individuals who use methamphetamine call for greater access to treatment, there is little known about methamphetamine use and treatment among individuals who are already engaged in outpatient substance use treatment [19–21]. There are currently no FDA-approved pharmacologic therapies for methamphetamine use disorder (MUD), though there have been single and multisite clinical trials conducted for a range of pharmacologic agents including antidepressants, anticonvulsants, stimulants and opioid antagonists [22, 23]. The behavioral therapy with the strongest evidence for MUD treatment is contingency management, a behavioral treatment that takes advantage of operant conditioning to promote therapeutic behaviors. It involves the provision of positive reinforcements, such as money or gift cards, for target behaviors such as drug abstinence. Multiple randomized controlled trials have demonstrated the efficacy of contingency management in reducing methamphetamine use and increasing treatment retention [24–27]. However, contingency management is not widely available and generally underutilized as a treatment [28]. Engagement in contingency management therapy is unknown for individuals with polysubstance use who are connected to care. Greater understanding of the specific needs of care-engaged individuals who use methamphetamine is necessary to guide harm reduction and effective treatment strategies.

This study aimed to characterize care-engaged individuals who use methamphetamine at a large academic medical center in Boston, Massachusetts across multiple social and medical domains. Following characterization of the cohort, we further sought to examine characteristics associated with injection methamphetamine use.

## Methods

### Study design and population

We performed a retrospective electronic medical record (EMR) review of patients at a large academic medical center in Boston, Massachusetts (48,000 admissions and 5,00,000 primary care visits annually) with ≥ 2 positive methamphetamine oral toxicology tests between August 1, 2019 and January 31, 2020. Oral fluid toxicology tests, conducted via liquid chromatography-tandem mass spectrometry, are highly specific for methamphetamines with a low likelihood of false positives [29]. We included all individuals with ≥ 2 positive methamphetamine oral toxicology tests to further increase specificity for individuals using methamphetamines more regularly [30]. We considered this patient population “care-engaged” as they receive integrated ambulatory and addiction care from the large health system through a combination of community health centers, primary care practices, infectious disease clinics, a low-threshold transitional addiction clinic, and peer recovery support. Medical records were reviewed and coded using information available up to July 1, 2020 to allow for a reasonable duration of medical follow up time for participants.

A list of data elements was created and refined iteratively (BB, JM). A data extraction instrument was then developed using REDCap (REDCap, Version 9.5.35) (BB, JM) [31]. Free text search terms and a search algorithm were appended to the REDCap instrument to standardize use between researchers (BB, JM).

Chart reviews were conducted between July 1 and September 1, 2020, using standardized terms to query the EMR via the free text search functionality (MYL). Areas of uncertainty were adjudicated between team members. Missing data points were included in the analysis and treated as missing at random. Following an initial review of all participant medical records, a 20% random sample was reviewed by a different team member, to reconcile inter-coder differences and check for missing data. Minor changes were made to the data instrument and all participants reviewed again to ensure fidelity to the updated instrument.

### Exposure and outcome definitions

We examined the sociodemographic, medical history and treatment, drug use, and high-risk behavior characteristics of study participants. Participants were assessed for lifetime history of characteristics unless otherwise specified.

Sociodemographic characteristics included: age, sex, sexual behavior, race/ethnicity, education, marital status, and lifetime history of homelessness and incarceration.

Medical characteristics included: substance use disorder (SUD) history including opioid, stimulant, benzodiazepine, alcohol, and hallucinogen use disorder; history of overdose from any drug; infection history including endocarditis, osteomyelitis, septic arthritis, superficial abscess, paraspinal abscess, and bacteremia; history of Hepatitis C virus; history of HIV; and psychiatric history including psychosis, depression, and suicidal ideation or attempt. An individual was considered to have a history of substance use if use was documented in ≥ 1 note, and considered to have a history of a SUD if explicit documentation of SUD appeared in ≥ 1 note or under an ICD-10-CM code.

Medical care characteristics included MUD treatment defined as contingency management or prescriptions for bupropion, naltrexone, rivastigmine, topiramate, modafinil, mirtazapine, or stimulant medications with an explicit indication for harm reduction or MUD treatment; past and current opioid use disorder (OUD) treatment including methadone, buprenorphine, and naltrexone; recent prescriptions of controlled substances written between 2019 and 2020; prescriptions of naloxone or pre-exposure prophylaxis for HIV prevention; and engagement in addiction specialty services including low-barrier-to-entry Bridge clinic, addiction psychiatry, a buprenorphine-prescribing primary care physician, office-based addiction treatment nurse, or recovery coach.

Drug use behaviors including route of methamphetamine use (injection, smoking, snorting, other) and high-risk behaviors (sex work and needle sharing) were also assessed.

### Statistical analysis

The overall prevalence of sociodemographic and medical characteristics were calculated for all participants. Participants were then stratified into two groups by their route of methamphetamine use: individuals who indicated methamphetamine use by injection and individuals who indicated methamphetamine use via smoking, snorting or other means. The groups were then compared across all variables via chi-square tests for categorical variables and Fisher’s Exact tests for continuous variables in STATA (version 16.1, STATACorp, College Station, TX), using a p value cut off of 0.05 for significant findings.

This study was approved by the Mass General Brigham Institutional Review Board.

## Results

### Sociodemographics

A total of 71 individuals had ≥ 2 positive oral toxicology tests between August 1, 2019 and January 1, 2020 and were included in this analysis (Table 1). The majority were male (80%), 66 non-Hispanic white (94%), and the median age was 36 (IQR = 30–42)]. Approximately 21% were males with documented history of sex with other males. A little under half (40%) graduated from high school while 12% graduated from college. Most were single (75%). Experiences of homelessness (83%) and incarceration (76%) were extremely prevalent.

**Table 1:** Characteristics of individuals using methamphetamine with and without evidence of injection methamphetamine use

	All participants n = 71	Injection meth use n = 48	No injection meth use n = 23	
	No. (%)	No. (%)	No. (%)	P value
Sociodemographics				
Age				
18–39	49 (69)	35 (73)	14 (61)	Pr(T < t) = 0.77
40–59	21 (30)	12 (25)	9 (39)	Pr(|T| > |t|) = 0.46
≥ 60	1 (1)	0 (0)	0 (0)	Pr(T > t) = 0.23
Sex				0.37
Male	56 (80)	39 (83)	17 (74)	
Men who have sex with men	14 (21)	10 (15)	4 (6)	0.98
Race/ethnicity				0.75
Non-Hispanic white	66 (94)	45 (96)	21 (91)	
Black	2 (3)	1 (2)	1 (4)	
Hispanic	2(3)	1 (2)	1 (4)	
Education				0.48
Some high school	10 (17)	8 (20)	2 (12)	
High school graduate	23 (40)	18 (44)	5 (29)	
Some college	18 (31)	11 (27)	7 (41)	
College graduate	7 (12)	4 (10)	3 (18)	
Marital status				0.30
Single	53 (75)	38 (79)	15 (65)	
Married	7 (10)	3 (6)	4 (17)	
Divorced/separated	11 (15)	7 (15)	4 (17)	
Homelessness	59 (83)	42 (88)	17 (73)	0.15
Incarceration	52 (76)	39 (81)	14 (64)	0.11
Medical characteristics				
SUD history				
Opioid use disorder	67 (94)	45 (94)	22 (96)	0.75
Stimulant use disorder	65 (92)	**47 (98)**	**18 (78)**	**0.01**
Benzodiazepine use disorder	29 (41)	**25 (52)**	**4 (17)**	**0.01**
Alcohol use disorder	30 (42)	22 (46)	8 (35)	0.38
Hallucinogen use disorder	1 (1)	1 (2)	0 (0)	0.49
Other	2 (3)	1 (2)	1 (4)	0.59
History of overdose	59 (84)	**44 (91)**	**16 (70)**	**0.02**
Infection history				
Endocarditis	10 (14)	9 (19)	1 (4)	0.10
Osteomyelitis	8 (11)	6 (13)	2 (9)	0.64
Septic arthritis	5 (7)	4 (8)	1 (4)	0.54
Superficial abscess	48 (68)	**36 (75)**	**12 (52)**	**0.05**
Paraspinal abscess	3 (4)	2 (4)	1 (4)	0.97
Bacteremia	18 (25)	13 (27)	5 (22)	0.63
Other	1 (1)	1 (2)	0 (0)	0.49
History of HCV	57 (80)	**45 (94)**	**12 (52)**	**< 0.01**
History of HIV	8 (11)	7 (15)	1 (4)	0.20
History of psychosis	30 (43)	23 (49)	7 (30)	0.14
History of depression	65 (92)	45 (94)	20 (87)	0.34
History of suicidal ideation/attempt	41 (58)	27 (56)	14 (61)	0.71
Care access				
Primary care provider (PCP)				0.09
PCP within our hospital system	31 (44)	17 (35)	15 (64)	
PCP outside our hospital system	34 (49)	26 (56)	7 (32)	
No PCP	5 (7)	4 (8)	1 (5)	
MUD treatment				
Pharmacologic	17 (24)	15 (32)	2 (9)	**0.03**
Contingency management	0 (0)	0 (0)	0 (0)	–
MOUD treatment				
Methadone	36 (51)	21 (44)	15 (65)	0.09
Buprenorphine	66 (93)	45 (94)	21 (91)	0.71
Naltrexone	11 (15)	**11 (23)**	**0 (0)**	**0.01**
No MOUD	6 (8)	4 (8)	2 (9)	0.96
Current MOUD	59 (83)	41 (85)	18 (78)	0.45
Prescription history				
Opioid	13 (18)	8 (17)	5 (22)	0.61
Stimulant	35 (49)	24 (20)	11 (48)	0.86
Benzodiazepine	24 (34)	16 (33)	8 (35)	0.90
Medical marijuana	3 (4)	1 (2)	2 (9)	0.2
History of naloxone prescription	60 (85)	42 (88)	18 (78)	0.31
History of PrEP treatment	20 (28)	16 (33)	4 (17)	0.16
Addiction specialty services				
Addiction consult team	49 (69)	36 (75)	13 (57)	0.11
Bridge clinic	57 (80)	41 (85)	16 (70)	0.11
West end clinic	23 (32)	14 (29)	9 (39)	0.40
Addiction psychiatry	43 (61)	31 (65)	12 (52)	0.32
Buprenorphine-prescribing PCP	18 (25)	11 (23)	7 (30)	0.50
OBAT RN	20 (28)	15 (31)	5 (22)	0.4
Recovery coach/peer support	56 (79)	40 (83)	16 (70)	0.18
Other	2 (3)	1 (2)	1 (4)	0.59
Drug use behaviors				
Drug use				
Fentanyl	59 (84)	**44 (94)**	**15 (65)**	**< 0.01**
Cannabis	64 (90)	45 (94)	19 (83)	0.14
Alcohol	64 (90)	**46 (96)**	**18 (78)**	**0.02**
Tobacco	66 (93)	45 (94)	21 (91)	0.71
Injection drug use	65 (92)	48 (100)	17 (74)	< 0.01
Route of methamphetamine use				
Injection	48 (68)	–	–	–
Smoking	22 (31)	15 (31)	7 (30)	0.95
Snorting	18 (25)	13 (27)	5 (22)	0.63
Other	2 (3)	1 (2)	1 (4)	0.59
High risk behaviors				
None	39 (55)	**21 (44)**	**18 (78)**	**< 0.01**
Sex work	9 (13)	**9 (19)**	**0 (0)**	**0.03**
Needle sharing	28 (39)	**26 (54)**	**2 (9)**	**< 0.01**
Other	0 (0)	0 (0)	0 (0)	–

Bolded rows are significantly different, p < 0.05

Significant p value = 0.5. Pharmacologic MUD treatment includes bupropion, naltrexone, rivastigmine, topiramate, modafinil, mirtazapine, or stimulant medications

*MOUD*  medications for opioid use disorder;   *MUD*   methamphetamine use disorder; *PrEP*  pre-exposure prophylaxis; *OBAT RN*   office-based addiction treatment nurse

### Medical and psychiatric co-morbidities

Participants had high prevalence of comorbid SUD including OUD (94%), stimulant use disorder (92%), alcohol use disorder (42%), and benzodiazepine use disorder (41%). The majority (84%) had experienced a non-lethal drug overdose. They had extensive histories of infections including Hepatitis C virus (HCV) (80%), superficial abscesses (68%), bacteremia (25%), endocarditis (14%), HIV (11%), osteomyelitis (11%), septic arthritis (7%), and paraspinal abscesses (4%). Co-occurring psychiatric conditions including depression (92%), suicidality (58%), and psychosis (43%) were common.

### Addiction care and treatment engagement

Most participants had received a prescription for naloxone (85%) and were currently on medication for opioid use disorder (83%) (MOUD). Nearly all participants (93%) had ever been treated with buprenorphine, with smaller proportions having ever been treated with methadone (51%), or naltrexone (15%) as MOUD. A sizable minority of participants had received recent prescriptions for stimulants (49%), benzodiazepines (34%), and opioid pain medications (18%), and 4% used medical cannabis via a certification in 2019 or 2020. Only 24% had documentation of pharmacologic treatment explicitly for their MUD, and none had received contingency management behavioral treatment. Only 28% received pre-exposure prophylaxis (PrEP) prescriptions to prevent HIV acquisition. Of those who received PrEP prescriptions, 40% were only given one-time prescriptions. A large proportion of individuals accessed addiction specialty services, such as a low-barrier-to-entry bridge clinic (80%), peer-support recovery coaching (79%), consultation from our academic medical center's addiction consult team (69%), and addiction psychiatry (61%). A smaller proportion received care from a registered nurse providing office-based addiction treatment (OBAT RN) (28%) or had a buprenorphine-prescribing PCP (25%).

### Drug use behaviors

Most individuals reported a history of using fentanyl (84%), cannabis (90%), alcohol (90%), and tobacco (93%). Nearly all reported injection use of any substance (92%) and some reported sharing needles at least once (39%). In our cohort, 13% reported engaging in sex work. Participants endorsed using methamphetamines via injection (68%), smoking (31%), and snorting (25%).

### Characteristics stratified by route of methamphetamine use

We compared the characteristics of individuals who inject methamphetamine with the characteristics of individuals who used methamphetamine by all other non-injection routes. Individuals who inject methamphetamine were significantly more likely to have a stimulant (98% vs. 78%; p = 0.01) and benzodiazepine use disorder (52% vs. 17%; p = 0.01) diagnosis compared to individuals who reported using methamphetamine exclusively via other routes. They were more likely to have a history of drug overdose (91% vs. 70%; p = 0.02), have HCV (94% vs. 52%; p < 0.01) or superficial abscesses (75% vs. 52%; p = 0.05), use fentanyl (93% vs. 65%; p = 0.02), use alcohol (96% vs. 78%; p = 0.02), share needles (54% vs. 9%; p < 0.01) and engage in sex work (19% vs. 0%; p = 0.03). Those who inject methamphetamine were more likely to have received MUD pharmacologic treatment (32% vs. 9%; p = 0.03) and naltrexone prescriptions (23% vs. 0%; p = 0.01).

No significant sociodemographic differences were found between these groups. Both had prevalent experiences of homelessness (88% vs. 73%; p = 0.15) and incarceration (81% vs. 64%; p = 0.11), depression (94% vs. 87%; p = 0.34), suicidal attempt/ideation (56% vs. 61%; p = 0.71), psychosis (49% vs. 30%; p = 0.14) and serious infections, including bacteremia (27% vs. 22%; p = 0.63), endocarditis (19% vs. 4%; p = 0.10), and osteomyelitis (13% vs. 9%; p = 0.64).

## Discussion

This retrospective cohort study is among the first to characterize outpatient care-engaged individuals who use methamphetamine at a large, urban academic center in the Northeast US, revealing a clinically and socially complex population with high prevalence of polysubstance use, intravenous drug use, psychiatric comorbidities, infectious complications, overdose, homelessness, and incarceration. Participants who reported injecting methamphetamine faced further risks, with a higher prevalence of drug overdose, HCV infection, fentanyl use, and sex work. Overall, we found a similarly elevated prevalence of adverse social determinants of health between people who used methamphetamine via injection versus other routes. Our cohort reflected characteristics previously associated with greater risk for MUD: male sex; limited college education; concomitant tobacco, cannabis, sedative use; and co-occurring mental illness. Notably, our study participants faced significant sociodemographic challenges and clinical complications similar to those noted in nationwide cohorts, despite their engagement in outpatient and specialty addiction care [21, 32].

Our cohort was notable for a high prevalence of polysubstance use, particularly co-occurring OUD and MUD in over 90% of participants. While nearly all participants were treated for their OUD with gold-standard MOUD, none of the 78% with documented stimulant use disorder had documented discussions of or referrals to the most effective evidence-based behavioral treatment for stimulant use disorder: contingency management [24–26, 33]. Although 24% of participants received some form of pharmacologic treatment for their MUD, all were prescribed for off-label use. While half of the participants in the study had received a stimulant prescription in the past year, the indication for the prescription was not always clear and may have included methamphetamine use harm reduction, attention deficit and hyperactivity disorder treatment, or other reasons. While there have been many medical trials involving participants with OUD, only one involved participants with comorbid OUD and MUD; the lack of medication trials in this growing population highlights an urgent need for further research [34].

Clinical trials have shown promising effects from stimulant agonist treatment (dexamphetamine or methylphenidate), mirtazapine, and combination bupropion and extended-release injectable naltrexone therapy, but these medications do not currently have FDA approval [22, 23, 35, 36]. Additionally, the modest effect of bupropion and extended-release naltrexone requires a prolonged period of opioid abstinence, which may be a barrier in a population of people with MUD and high likelihood of comorbid OUD.

Taken together, our results suggest the need to screen care-engaged individuals with methamphetamine use for concomitant OUD, and vice versa, and highlight that contingency management is an underutilized treatment in this population. Despite its strong evidence base, contingency management is the least commonly delivered behavioral intervention for substance use disorders, owing to philosophical and durability concerns amongst providers and economic challenges with limited CM reimbursement through insurance [28]. Given the extremely limited availability of CM treatment across the US, rapid development and implementation of such programs is necessary [33]. The Veterans Administration’s highly successful nationwide implementation of CM across all its outpatient substance use treatment clinics serves as a model for integrating CM into addiction care [37].

Moreover, in the context of limited available treatment options for MUD and the high prevalence of polysubstance use, evidence-based harm reduction practices that reduce mortality and morbidity across all types of substance use is critical. Individuals who use injection methamphetamine may particularly benefit from increased access to naloxone, fentanyl test strips, safer sex kits and sterile injection supplies, given their higher prevalence of overdose, Hepatitis C virus (HCV), and engagement in high-risk sex practices. Additionally, nearly half of the participants who received PrEP were not continued long-term on preventative HIV treatment. Due to increased HIV prevalence, sex work, and injection practices among people who use drugs in Massachusetts, providing maintenance PrEP prescriptions coupled with routine sexually transmitted infection testing is a critical step in mitigating HIV spread [38]. To address the multifaceted needs of individuals with SUDs, co-located multidisciplinary care systems providing SUD treatment, infectious disease care, behavioral treatment, and harm reduction resources have been developed in primary care, Human Immunodeficiency Virus (HIV) specialty care, opioid treatment program, and transitions clinic settings [39]. Inclusion of CM would be appropriately placed within the context of other models of integrated addiction care. Finally, the high prevalence of homelessness and incarceration, despite relatively strong treatment engagement, highlight the need for affordable housing and drug law reform alongside any clinical or public health intervention.

This study must be viewed in light of its limitations. First, the study—which included a predominantly white and care-engaged participant population—was conducted at a single large urban academic health center and may not be generalizable to a wider population. Second, the relatively small sample size provides limited power to capture statistically significant differences between participants who inject methamphetamine versus use by other routes. Third, this was a retrospective study conducted by EMR review and answers to certain questions (for example, route of drug use) are limited by documentation variability and participants’ social desirability biases. With regards to the self-reported route of use, we believe the result would be biased towards no difference between the groups and may reflect an underestimate of individuals who inject methamphetamine. Additionally, because this study relied on retrospective EMR review and healthcare provider documentation, we were limited in our ability to capture care outside our medical system and accurately account for individuals’ self-identified gender and sexual orientations in our analyses. Prospective studies of people who use methamphetamine, coupled with explanatory qualitative interviews, are warranted.

## Conclusions

Individuals in our study had high prevalence of polysubstance use, particularly concomitant methamphetamine and opioid use. Our findings are concordant with findings from nationwide cohorts demonstrating significant sociodemographic challenges and clinical complications among individuals who use methamphetamine. Individuals who are well connected to substance use treatment for their OUD are still likely to be undertreated for their MUD and would benefit from greater access to contingency management treatment, harm reduction resources, and resources to address adverse social determinants of health.

## Data Availability

All data generated and datasets used during the current study will be available from the corresponding author on reasonable request.
